# Dual-Wave ZnO Film Ultrasonic Transducers for Temperature and Stress Measurements

**DOI:** 10.3390/s24175691

**Published:** 2024-09-01

**Authors:** Wentao Liu, Longlong Shan, Zhongliang Lin, Binghua Wan, Bin Yang, Xiaomei Zeng, Bing Yang, Vasiliy Pelenovich

**Affiliations:** 1 Aerospace Precision Products Inc., Ltd., Tianjin 300300, China; 2 Institute of Technological Sciences, Wuhan University, Wuhan 430072, China; 3 School of Power & Mechanical Engineering, Wuhan University, Wuhan 430072, China; toyangbing@whu.edu.cn

**Keywords:** columnar structure, high-temperature ultrasonic transducer, longitudinal and transverse waves, magnetron sputtering, thin-film ZnO

## Abstract

ZnO film ultrasonic transducers for temperature and stress measurements with dual-mode wave excitation (longitudinal and shear) were deposited using the reactive RF magnetron sputtering technique on Si and stainless steel substrates and construction steel bolts. It was found that the position in the substrate plane had a significant effect on the structure and ultrasonic performance of the transducers. The transducers deposited at the center of the deposition zone demonstrated a straight columnar structure with a *c*-axis parallel to the substrate normal and the generation of longitudinal waves. The transducers deposited at the edge of the deposition zone demonstrated inclined columnar structures and the generation of dominant shear or longitudinal shear waves. Transducers deposited on the bolts with dual-wave excitation were used to study the effects of high temperatures in the range from 25 to 525 °C and tensile stress in the range from 0 to 268 MPa on ultrasonic response. Dependencies between changes in the relative time of flight and temperature or axial stress were obtained. The dependencies can be described by second-order functions of temperature and stress. An analysis of the contributions of thermal expansion, strain, and the speed of sound to changes in the time of flight was performed. At high temperatures, a decrease in the signal amplitude was observed due to the decreasing resistivity of the transducer. The ZnO ultrasonic transducers can be used up to temperatures of ~500 °C.

## 1. Introduction

Bolts are the most widely used fasteners in various mechanical and structural components. During operation, high temperatures and stress can change the bolt preload, affecting the safety of the components. Therefore, it is important to monitor the bolt preload and temperature [1]. The simplest bolt tightening force measurement can be conducted using the torque wrench method [2]. The method is based on the linear relationship between the bolt preload and applied torque. However, a poorly controlled coefficient of friction between the bolts and mechanical components and different types of lubricating materials result in high experimental uncertainty in the load measurement of ~25% [1]. Another method, the Fiber Bragg Grating (FBG) method, uses a fiber-optic sensor, which is a wavelength-dependent reflector with a periodic refractive index structure. The strain or temperature expansion in the bolt changes the period of the refractive index structure and, therefore, the wavelength of the reflected light [3,4]. In the method, the FBG sensor is embedded into the bolt. This, however, requires an additional machining and reduces the strength of the fastener. Therefore, the nondestructive health monitoring of fasteners, such as that based on the acoustoelastic effect, should be used [5,6,7]. In this method, the stress and temperature in the bolt can be measured by changes in the pulse-echo time of flight Δ*t* due to changes in the bolt length and speed of sound:

(1) $$\Delta t=t-t_{0}=\frac{2l}{v}-\frac{2l_{0}}{v_{0}}$$

where *l* and *v* are the length of the bolt and speed of sound under a certain axial stress *σ* and temperature *T*, and *l*_0_ and *v*_0_ are the length and speed at initial temperature *T*_0_ without stress (*σ*_0_ = 0); the coefficient 2 describes the forward and backward propagations of the wave.

If the temperature change Δ*T = T − T*_0_ and axial stress *σ* are small enough, the length *l* of the bolt can be described as a Taylor series in the linear approximations:

(2) $$l=l_{o}+l_{o}\alpha \Delta T=l_{o}(1+\alpha \Delta T)\;\mathrm{and}\;l=l_{o}+l_{o}E^{-1}\sigma =l_{o}(1+E^{-1}\sigma )$$

where α is the coefficient of thermal expansion (CTE), and *E* is the Young’s modulus of the bolt material. The same can be described for the speed of sound *v*:

(3) $$v=v_{o}+v_{o}\beta \Delta T=v_{o}(1+\beta \Delta T)\;\mathrm{and}\;v=v_{o}+v_{o}C\sigma =v_{o}(1+C\sigma )$$

where *β* and *C* are coefficients describing the effects of temperature and stress, respectively.

If there are both a temperature change and axial stress, the effects can be summed:

(4) $$l=l_{o}(1+\alpha \Delta T+E^{-1}\sigma )\;v=v_{o}(1+\beta \Delta T+C\sigma )$$

Therefore, the total change in the time of flight can be written as:

(5) $$\Delta t=\frac{2l_{o}(1+\alpha \Delta T+E^{-1}\sigma )}{v_{o}(1+\beta \Delta T+C\sigma )}-\frac{2l_{0}}{v_{0}}$$

or for the relative change in the time of flight,

(6) $$\frac{\Delta t}{t_{0}}=\frac{1+\alpha \Delta T+E^{-1}\sigma }{1+\beta \Delta T+C\sigma }-1\approx (\alpha -\beta )\Delta T+(E^{-1}-C)\sigma$$

Therefore, the temperature and stress in the bolt can be calculated using the measured relative change in the time of flight using Formula (6). The material parameters α, β, *E*, and *C* can be determined in the calibration procedures using the known values of the temperature change and axial stress. Formula (6) is applicable for both longitudinal and shear ultrasonic waves. Due to the different speeds of sound of these waves, it is necessary to distinguish the parameters β_L_, *C_L_* and β_S_, *C_S_* of these two waves. 

The stress measurement is complicated by the fact that the total length of the bolt is not equal to the clamping length (the length under stress), which depends on the position of the bolt nut. A nonhomogeneous stress field in the bolt results in a complex dependence between the time of flight and speed of sound, and Formula (6) needs to be corrected [6,7,8].

Among numerous piezoelectric materials, ZnO is a promising material that has attracted attention due to its high piezoelectric strain constant *d*_33_ among tetrahedral semiconductors and wide bandgap of 3.37 eV, which is suitable for optoelectronic applications [9,10]. In its bulk form, ZnO (0001) demonstrates a piezoelectric constant *d*_33_ of ~10 pC/N [11,12]. The deposition of the transducers in film form simplifies the problems of adhesion between the piezoelectric transducer and the substrate, which greatly improves the durability of the sensor. The hexagonal structure of ZnO (space group P63mc) results in the relative ease of deposition of the thin films oriented along the *c*-axis perpendicular to the substrate normal using magnetron sputtering techniques [13,14,15,16]. Since the *c*-axis is polar, the transducer can generate longitudinal waves. The transducer with an inclined columnar structure can generate both longitudinal and shear waves [17], which makes it a convenient tool in stress and temperature measurements. 

In this study, we use the radio frequency (RF) magnetron sputtering technique to deposit ZnO film transducers on bolt heads and other substrates. Changing the position of the sample relative to the target, we control the inclination of the ZnO columnar structure, and, by this way, the mode of wave generation of the transducer. Next, we study the effects of temperature and stress in the bolt on ultrasonic response.

## 2. Materials and Methods

**Figure 1:**
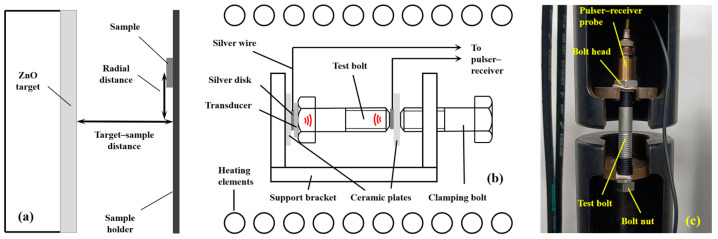
Schematic diagrams of the deposition geometry (**a**) and bolt fixture for high temperature measurement (**b**); M14 bolt in the fixture for tensile stress measurement (**c**).

Zinc oxide piezoelectric film transducers were deposited onto Si (100) and stainless steel (AISI 304, Shuangmeng Nonferrous Metal Products Co., Ltd., Zhuzhou, China) substrates and on the heads of bolts (M14 × 109, Jinchao Hardware Products Co., Ltd., Xinghua, China) using the RF magnetron sputtering technique. Before the deposition, the bolt head surface was mechanically polished, and all samples were ultrasonically cleaned with acetone, alcohol, and deionized water. The transducers consisted of three layers (from bottom to top): a ZnO piezoelectric layer, amorphous Si oxide layer, and Cr top electrode layer with a diameter of 3 mm. The bottom electrodes were the substrates and bolts themselves. The deposition of the Si oxide layer is to increase the electrical strength of the sensor, as an unprotected ZnO thin-film transducer, due to its small thickness, is prone to breakdown by the spikes generated by the pulser–receiver. As targets ZnO, Si, and Cr disks with a diameter of 150 mm and purity of 99.99% were used. The optimized deposition parameters from our previous studies are listed in Table 1 [18,19]. The cross-section deposition geometry is shown in Figure 1a. The variable parameter in this study was the radial distance, which was adjusted by shifting the substrate in the sample holder plane.

The surface and cross-section morphologies of the deposited transducers were studied using the scanning electron microscope (SEM) TESCAN MIRA3 (TESCAN, Brno-Kohoutovice, Czech Republic) equipped with an energy dispersive spectrometer (EDS) Aztec Energy-X-Max 20 (Oxford Instruments, High Wycombe, UK) for element composition analysis. The phase composition and crystal structure were studied using the X-ray diffractometer (XRD) Tongda TDM-10 (Dandong Tongda Science and Technology Co., Ltd., Dandong, China) equipped with a Cu K_α_ (λ = 0.154 nm) radiation source.

The ultrasonic pulse-echo tests were carried out with a JSR Ultrasonics DPR300 ultrasonic pulser–receiver. To generate ultrasonic waves, negative voltage spikes with an amplitude of 50 V and length of 50 ns were applied to the transducer. The reflected ultrasonic waves were detected with the same transducer and converted to the electrical signal. More details on the measurement procedures can be found in our previous studies [20,21].

In situ high-temperature pulse-echo measurements were carried out in an electric furnace in an air atmosphere, with gradual increases in the temperature in the range from 25 to 525 °C with a speed of 2 K/min. The bolt fixture schematic for the temperature measurement is shown in Figure 1b. The steel support bracket and clamping bolt were used to fix the test bolt with the deposited ZnO sensor, two insulating ceramics plates, and two silver disc electrodes with silver wires in a stack. 

Pulse-echo measurements under tensile stress were carried out by using a stretching machine, Shimadzu AGX-V. The test bolt installed in the fixture is shown in Figure 1c. The axial loads were 0, 10, 20, 30, and 40 kN. The loading speed was 1 kN/s. The axial load range was chosen to maintain the stress in the bolt within the linear range of the stress–strain curve. In this study, we do not consider the correction of the clamping length in Formula (6). In order to minimize its impact, we place the nut at the limit position of the bolt, so that the clamping length is 90% of the total length of the bolt.

## 3. Results and Discussion

### 3.1. Transducer Structure and Ultrasonic Response

**Figure 2:**
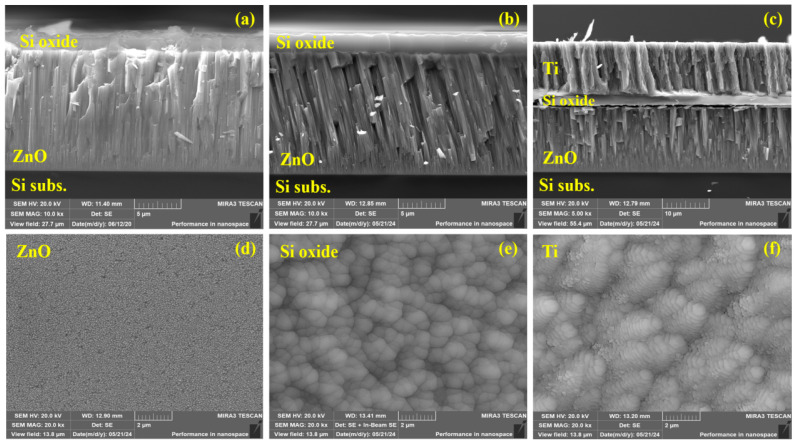
SEM cross-section images of the ZnO transducers deposited at the center (**a**) and edge (**b**) of the deposition zone; cross-section image of the sensor with the top electrode (**c**); the surface SEM images of the ZnO (**d**), Si oxide (**e**), and Ti (**f**) layers.

In Figure 2a,b, the SEM cross-section images of ZnO transducers deposited on Si substrates at the center and edge of the deposition zone are shown. The transducer deposited in the center has a columnar structure with columns that are nearly perpendicular to the substrate normal, Figure 2a. The transducer deposited at the edge has a tilted columnar structure with inclination angle of ~15°, Figure 2b. The formation of the tilted columnar structure can be explained by the shadowing effect [22]. This effect only becomes apparent when the sputtered target material arrives to the substrate surface at an oblique angle, which is realized at the edge of the deposition zone. This condition and the low surface diffusion of the deposited particles result in the formation of a tilted columnar structure. The thickness of the transducer layers is ~15 μm. Surface EDS analysis of the transducers deposited on Si substrate revealed an average Zn:O atomic ratio of 1:1.02, which is very close to the stoichiometric ratio of 1:1. The cross-section of the complete structure of the sensor with the Ti electrode is shown in Figure 2c. The surface SEM images of the ZnO, Si oxide, and Ti layers at the same scale are shown in Figure 2d, Figure 2e, and Figure 2f, respectively. The particulate morphology in Figure 2d represents the tops of the ZnO columns.

**Figure 3:**
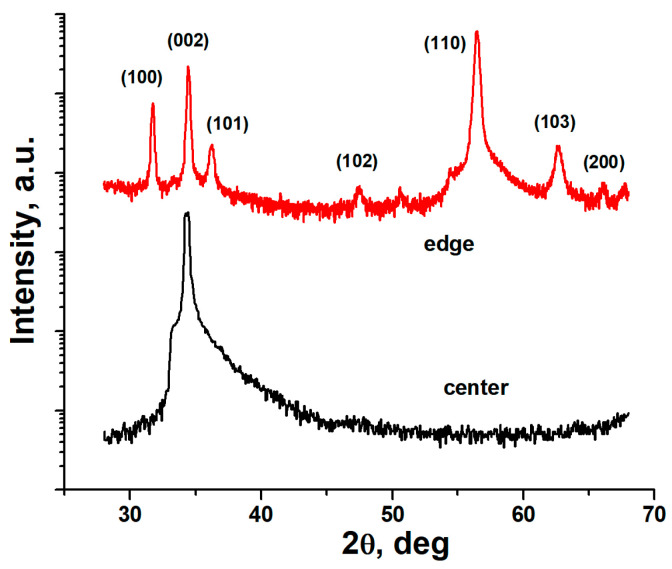
XRD patterns of the ZnO transducers deposited on SS substrates at the center and edge of the deposition zone.

XRD patterns of the ZnO transducers deposited on SS substrates at the center and edge of the deposition zone are shown in Figure 3. The transducer deposited in the center is grown using the (002) preferred orientation, and no other XRD peaks are observed, which means that the *c*-axis of the crystal cell is oriented along the columns. The transducer grown at the edge revealed strong (100), (110), and (002) reflexes, and a few others with a lower intensity. This result means that the *c*-axis of the columns is no longer perpendicular to the substrate plane; that is, the *c*-axis is tilted. The obtained results are in agreement with the SEM data.

**Figure 4:**
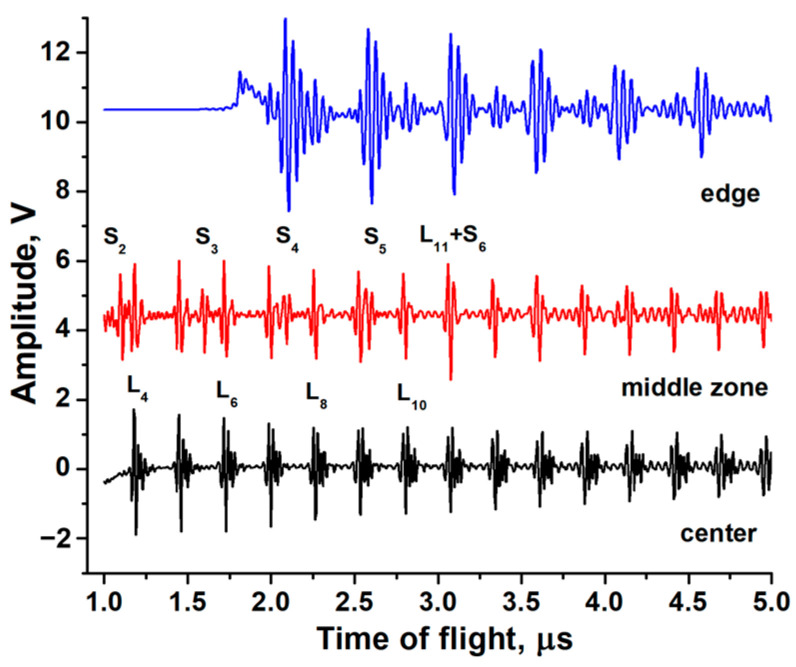
Pulse-echo responses of transducers deposited on the SS substrates at different positions in the deposition zone.

The pulse-echo responses of transducers deposited at different positions in the deposition zone are shown in Figure 4. The transducer deposited in the center with the *c*-axis perpendicular to the substrate surface generates pure LWs. Strong SWs are generated by the transducer with a tilted columnar structure. The most interesting situation for transducers deposited in the middle region is when both LWs and SWs are observed simultaneously. Therefore, the results show that the position of the sample in the deposition zone can effectively control the relative amplitudes of the LWs and SWs.

The obtained results can be understood using a matrix representation of the piezoelectric properties of ZnO transducer. The ultrasonic wave can be described by the strain values [23]:

(7) $$S_{i}=d_{ki}E_{k}=d_{1i}E_{1}+d_{2i}E_{2}+d_{3i}E_{3},$$

where *S_i_*, *i* = 1, …, 6 are the strain tensor components; the *S*_1_, *S*_2_, and *S*_3_ components describe the LW; the *S*_4_, *S*_5_, and *S*_6_ components describe the SW; *E_k_* is the electric field vector component; and *d_ki_* is the piezoelectric strain constant, for which the ZnO can be written as [12]:

(8) $$d_{ki}=\left(\begin{array}{cccccc} 0 & 0 & 0 & 0 & d_{15} & 0\\ 0 & 0 & 0 & d_{15} & 0 & 0\\ d_{31} & d_{31} & d_{33} & 0 & 0 & 0 \end{array}\right)=\left(\begin{array}{cccccc} 0 & 0 & 0 & 0 & -8.3 & 0\\ 0 & 0 & 0 & -8.3 & 0 & 0\\ -5.1 & -5.1 & 12.3 & 0 & 0 & 0 \end{array}\right)$$

In order to generate the ultrasonic wave, we apply a voltage spike to the transducer. If the transducer is grown in the center, its *c*-axis is perpendicular to the substrate surface and parallel to the applied electric field *E*. Considering that the *c*-axis belongs to the *z*-axis of the coordinate system, the electric field components are *E*_1_ = *E*_2_ = 0 and *E*_3_ ≠ 0. Then. the strain components can be rewritten as:

(9) $$S_{i}=d_{3i}E_{3}\;\mathrm{or}\;S_{1}=S_{2}=d_{31}E_{3},\;S_{3}=d_{33}E_{3},\;\mathrm{and}\;S_{4}=S_{5}=S_{6}=0.$$

It can be seen that for a straight columnar structure, only LWs can be generated. The transducer with an inclined columnar structure has *E_i_* ≠ 0 for all *i* and, according to Formula (7), all strain components *S_i_* are nonzero. Therefore, both LWs and SWs can be generated.

### 3.2. Temperature and Tensile Stress Measurements

**Figure 5:**
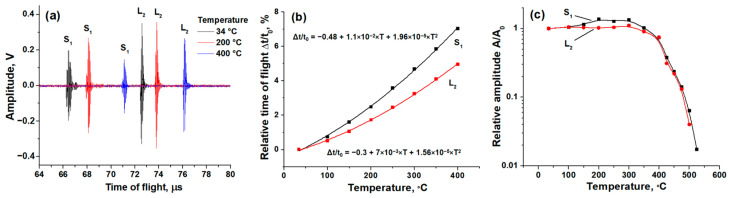
Pulse-echo responses of transducers deposited on head of M14 bolt and measured at different temperatures (**a**), temperature dependencies of the relative TOF (**b**), and temperature dependencies of the relative amplitudes (**c**) of second LW and first SW echoes.

The transducers with dual LW and SW excitation deposited on the heads of M14 bolts were used to study the effects of temperature and stress on ultrasonic response. In Figure 5a, the ultrasonic responses at 34, 200, and 400 °C are shown. At higher temperatures, an increase in the TOFs and decrease in the pulse amplitudes of both LWs and SWs are observed. Temperature dependencies of the relative TOFs of LWs and SWs are shown in Figure 5b. Both of these dependencies can be approximated well by using a second-order (parabola) function of temperature, shown in Figure 5b. The increase in the TOF can be explained by the linear thermal expansion of the bolt (increase of the bolt length *l*) and by a decrease in the speed of sound *v*. The relative change in the TOF due to thermal expansion can be written by using Formula (6) as:

(10) $${\left(\frac{\Delta t}{t_{0}}\right)}_{CTE}=\alpha \Delta T,$$

where Δ*T* is the temperature interval, and α = 11.8 × 10^−6^ K^−1^ [24] is the CTE of pure iron at 25 °C. It should be noted that the CTE α itself is a function of temperature; moreover, Formula (6) is applicable for small temperature ranges Δ*T*. Using Formula (10), we can estimate the relative change in the TOF in the temperature range from 34 to 150 °C to be 0.0014 or 0.14%. However, the experimental TOF change in the same temperature range is ~1.6%, which is one order of magnitude higher than the calculated value. Therefore, it can be concluded that the main contribution to the change in the TOF is the decrease in the speed of sound. Using Formula (6), it can be written as:

(11) $${\left(\frac{\Delta t}{t_{0}}\right)}_{v}=-\beta \Delta T$$

However, to estimate the experimental data, it is convenient to use a formula for calculating the speed of sound [25]: $v\sim \sqrt{E/\rho }$, where *E* is the Young’s modulus, and ρ is the density. Then, due to the small thermal expansion, we can consider only the temperature change in *E*, and the relative change in the TOF can be rewritten by using the initial formula (Formula (1)) as:

(12) $${\left(\frac{\Delta t}{t_{0}}\right)}_{v}=\sqrt{\frac{E_{0}}{E_{T}}}-1,$$

where *E*_0_ and *E_T_* are the Young’s modulus at initial and high temperatures, respectively. Then, using experimental data on the deterioration of the Young’s modulus within the temperature range from 34 to 400 °C, with *E*_400_/*E*_34_ of ~0.9 [26], we can obtain a relative change in the TOF of 5.4%, which is in good agreement with our experimental result of 4.9% for LWs.

In Figure 5c, the temperature dependencies of the pulse amplitudes of LWs and SWs are shown. The amplitudes are nearly constant in the temperature range from 34 to 350 °C. In the range from 350 to 525 °C, they decrease exponentially by almost two orders of magnitude. In principle, these dependencies can also be used to measure the temperature but only within this narrow high-temperature range. The observed decrease in the amplitude limits the use of ZnO transducers to temperature of ~500 °C and can be explained by the temperature dependence of the resistivity of the ZnO layer due to its not-large-enough band gap, i.e., due to the effective generation of intrinsic charge carriers at high temperatures. At higher temperatures, due to the semiconducting properties of the layer, its resistivity decreases, which results in an increase in the leakage current and shunting of the input response measurement circuits of the pulser–receiver.

**Figure 6:**
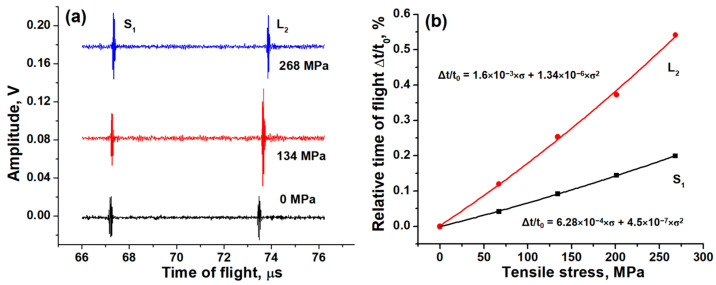
Pulse-echo response of transducers deposited on M14 bolt head and measured under different tensile stresses at room temperature (**a**); stress dependencies of the relative TOF of second LW and first SW echoes (**b**).

The same dual-wave transducers deposited on M14 bolts were tested for tensile stress measurements. In Figure 6a, the pulse-echo responses under different stresses are shown. Higher stress results in bolt deformation (elongation) and changed in the speed of sound. The effect of deformation on the TOF can be described by using Formula (6):

(13) $${\left(\frac{\Delta t}{t_{0}}\right)}_{def}=\frac{\sigma }{E},$$

where σ is the tensile stress in the bolt, and *E* is the Young’s modulus, which is equal to 210 GPa [27]. The estimation of the relative change in the TOF under a stress of 67 MPa gives 0.032%. The result is close to the experimental change in the TOF of 0.042% for SWs, Figure 6b. This is in agreement with the fact that the tensile stress has a smaller effect on the SW’s speed of sound [28]. This effect is much stronger on the LW’s speed of sound. Therefore, the experimental TOF delay of 0.112% for LWs should be explained by both the deformation of the bolt under stress and the decrease in the speed of sound.

A more detailed discussion of the dependence of the speed of sound on temperature and stress requires the consideration of the phonon model of the metal crystal structure. According to the Bohm–Staver formula [29], the speed of sound v in the metal can be expressed using the radius of the Fermi sphere of the free electrons *k_F_*:

(14) $$v=const\cdot k_{F},$$

where the constant contains the atomic number *Z* and the masses of the free electron and metal ion. The *k_F_* can be represented by the concentration of free electrons *n*:

(15) $$n=\frac{k_{F}^{3}}{3\pi^{2}}.$$

When the temperature of the bolt increases or it is under a tensile stress, and its density decreases due to thermal expansion or tensile strain, respectively. The decrease in the material density means a decrease in the free electron density; therefore, the electron concentration n decreases, and according to Formulas (14) and (15), the speed of sound also decreases.

In this study, we approximate the experimental temperature and stress dependencies by functions of the second order (see Figure 5b and Figure 6b), whereas the theoretical description uses a less accurate linear equation (Formula (6)). We can modify Formula (6) by adding second-order terms in the Taylor series as:

(16) $$\frac{\Delta t}{t_{0}}=(\alpha -\beta )\Delta T+(E^{-1}-C)\sigma +D\Delta T^{2}+E\sigma^{2}+F\Delta T\sigma$$

where *D*, *E*, and *F* are material constants, which are unknown and should also be found in calibration procedures. Although Formula (16) is more accurate, the linear equation (Formula (6)) is more convenient for practical use. Therefore, in order to minimize the contributions of the second-order terms, in the calculation, we have used narrow ranges of temperature and stress.

## 4. Conclusions

ZnO film ultrasonic transducers were deposited using the reactive RF magnetron sputtering technique on Si and stainless steel substrates and construction steel bolts. By adjusting the position of the substrate in the chamber relative to the target, the microstructure of the ZnO transducer and, therefore, its ultrasonic performance can be controlled. The transducers deposited at the center of the deposition zone demonstrate a straight columnar structure and the generation of LWs. At the edge of the deposition zone, transducers with an inclined columnar structure that generate the dominant SWs can be deposited. With a proper position adjustment, transducers with dual-mode wave excitation (LW and SW) can be fabricated. Transducers deposited on the M14 bolts with dual-wave excitation were used to study the effects of high temperatures in the range from 25 to 525 °C and tensile stress in the range from 0 to 268 MPa on ultrasonic response. The relative TOF dependencies on temperature and axial stress were obtained. Both dependencies are approximated well using second-order functions. For the temperature dependencies, the contribution of linear thermal expansion to the TOF change is only 10% of the measured values of LWs and SWs. Therefore, the main effect on the TOF correspond to the decrease in the speed of sound due to a decrease in the Young’s modulus at a high temperature. The axial stress affects the length of the bolt and the speed of sound of LWs. The effect on the speed of sound of SWs is not observed. The ZnO ultrasonic transducers can be used up to temperatures of ~500 °C. At high temperatures, a decrease in signal amplitudes is observed due to the decreasing resistivity of the ZnO transducer.

## Figures and Tables

**Table 1:** Deposition parameters of ZnO film transducers.

Deposition Parameter	Transducer	Protective Layer	Electrode
Target	ZnO	Si	Cr
Target–sample distance, mm	80	50	70
Radial distance range, mm	0–50		
RF power, W	700		
Temperature, °C	150		
Base pressure, Pa	5 × 10^−3^		
Working pressure, Pa	1.5		
Gas atmosphere, flow ratio	Ar:O_2_ = 1:1		Ar
Deposition time, h	4	2	1
Thickness of the layer, μm	~15	~2	~10

## Data Availability

The original contributions presented in the study are included in the article, further inquiries can be directed to the corresponding authors.
